# Example of Intrafamilial Clinical Polymorphism in a Family with Osteogenesis Imperfecta

**DOI:** 10.3390/genes16050475

**Published:** 2025-04-23

**Authors:** Varvara A. Galkina, Tatyana A. Vasilyeva, Inna S. Tebieva, Zolina K. Getoeva, Andrey V. Marakhonov, Vitaly V. Kadyshev, Sergey I. Kutsev, Rena A. Zinchenko

**Affiliations:** 1 Research Centre for Medical Genetics, 115522 Moscow, Russia; vgalka06@rambler.ru (V.A.G.); marakhonov@gmail.com (A.V.M.); vvh.kad@gmail.com (V.V.K.); kutsev@mail.ru (S.I.K.); renazinchenko@mail.ru (R.A.Z.); 2 North-Ossetian State Medical Academy, 362019 Vladikavkaz, Russia; tebinna@mail.ru; 3 Republican Children’s Clinical Hospital, 362003 Vladikavkaz, Russia; 4 Center for the Protection of Motherhood and Childhood in Sochi, 354057 Sochi, Russia; zalyna@yandex.ru

**Keywords:** osteogenesis imperfecta, clinical polymorphism, genetic heterogeneity, *COL1A1*

## Abstract

**Background/Objectives**: According to the International Classification of Hereditary Skeletal Diseases (2019), osteogenesis imperfecta (OI) is classified as a disorder resulting from impaired formation of the cortical layer density of diaphyses and metaphyseal modeling. OI comprises a heterogeneous group of genetic diseases, with most cases inherited in an autosomal dominant manner, while others follow autosomal recessive or X-linked recessive inheritance patterns. Accurate DNA testing is essential for precise medical and genetic counseling, ensuring reliable prognostic assessments for patients’ descendants and siblings. As part of a medical genetic study of the population of the Republic of the North Ossetia Alania, specifically in the Mozdok district, specialists from the Laboratory of Genetic Epidemiology at the Research Centre for Medical Genetics (RCMG) examined a family with 13 affected individuals with OI across four generations. **Methods**: A comprehensive clinical assessment was performed, followed by molecular genetic analysis using whole-exome sequencing (WES). Segregation analysis within the family was conducted via Sanger sequencing. **Results**: Clinical evaluation suggested a diagnosis of OI, which was subsequently confirmed by genetic testing. The severity and spectrum of symptoms varied considerably among affected family members and were influenced by age and specific nuclear family lineage. Molecular analysis in the proband identified a heterozygous pathogenic variant in the *COL1A1* gene variant (c.1243C>T, p.(Arg415*)), confirming a diagnosis of OI type IV. The variant was found to co-segregate with the disease within the family. **Conclusions**: Molecular diagnosis enabled precise risk assessment for affected offspring in family members with mild phenotypic manifestations. Additionally, pediatric patients were referred for standard bisphosphonate therapy to manage the condition effectively.

## 1. Introduction

Among the hereditary systemic skeletal diseases characterized by decreased density of the cortical layer of the diaphyses and impaired formation of the metaphyses, osteogenesis imperfecta (OI) is the most common and best studied form [1]. The prevalence of OI is approximately 4–20 per 100,000 [2,3,4,5].

The primary clinical manifestations of OI include bone fragility, which often leads to fractures occurring with minimal or no trauma. Affected individuals may also exhibit short stature and a reduced growth rate during childhood. In severe forms of OI, recurrent fractures can result in progressive limb and spinal deformities. Radiographic evaluation typically reveals several characteristic abnormalities. These include the presence of Wormian bones (small accessory bone plates) in the skull, cortical bone thinning in the diaphyses, generalized osteoporosis, and widened metaphyses. A key diagnostic radiographic feature is the biconcave (“codfish”) deformity of the vertebral bodies in the thoracolumbar spine. In some cases, this vertebral deformity may be the sole detectable clinical sign of OI [6,7]. Additional symptoms of OI include conductive hearing loss (otosclerosis), blue sclera (resulting from thinning of the connective tissue covering the eyeball), dentinogenesis imperfecta (DI, hypoplasia, enamel dysplasia, caries), triangular facies, broad forehead/macrocephaly, basilar invagination, connective tissue dysplasia (scoliosis, kyphosis, hypotonia and fragility of the skin, subluxations, hernias, cardiovascular anomalies), eye anomalies (glaucoma, astigmatism, myopia, keratoconus).

There are five clinical types of OI [1,3,8]. OI type I is characterized by a mild course, absence of bone deformities, normal or short stature, blue sclerae, and absence of DI. OI type II is characterized by multiple rib and long bone fractures at birth, severe deformities, low bone density on skull radiographs, and dark sclerae. OI type III’s main features are extremely short stature, triangular facies, severe scoliosis, grayish sclerae, and DI. OI type IV patients have moderately short stature, varying degrees of scoliosis, and blue, grayish, or white sclerae and may have DI. Type V is characterized by white sclerae, absence of DI, short stature, from mild to moderate, moderate deformities, metaphyseal dysplasia at birth with dislocation of the radial head, calcification of the interosseous membranes of the forearm and cnemis of the leg, and/or hyperplastic callus formation [8].

The manifestations and severity of osteogenesis imperfecta (OI) can vary significantly, ranging from intrauterine fractures and perinatal mortality to very mild cases without fractures throughout a patient’s lifetime. Even among affected individuals within the same family, the clinical presentation of OI may differ. Most patients (83.5%) [9] with OI have clinical types I–IV, which are caused by heterozygous pathogenic variants in the *COL1A1* or *COL1A2* genes. These genes encode the α-1 and α-2 chains of type I collagen. To date, over 1500 dominantly inherited pathogenic variants in these genes have been reported [10]. Dominant mutations in *COL1A1/2* are responsible for 90% of OI cases. Recently, several rare OI forms were characterized, they link to pathogenic variants in over 20 other genes; clinically, the rare forms of OI are not distinguished from traditional classification forms [11,12] (OMIM Phenotypic Series: PS166200) [5,9,13]. Hereditary syndromes characterized by low bone mineral density combined with OI symptoms have also been described [1,7].

OI presents one of collagenopathies. OI results from the main bone and tendon organic component collagen 1 function deficit developing due to its decreased quantity or abnormal structure. Other genes could influence collagen folding, post-translational modifications, processing, bone mineralization, and the differentiation of osteoblasts. Their disfunction also leads to the OI development [14]. At the cell level, OI impacts normal procollagen 1 and collagen 1 synthesis and processing in osteoblasts, as well as transport into the intercellular matrix. OI affects osteoblast and osteoclast maintenance and extracellular matrix content and mineralization [14]. OI also disrupts the balance in mesenchymal precursor differentiation into the osteoblasts and adipocytes. OI is characterized by thin cortex and its microcracks, bone fragility, multiple fractures, skeletal deformities, reduced bone mineral density, and impaired bone mineralization [14,15].

Selecting a gene for searching for a causative genetic variant based on the clinical picture, especially in a single case of the disease in a family, is often difficult, due to similar phenotypic manifestations or partial manifestation of symptoms in a number of patients. Next-generation sequencing (NGS) testing could be used as a first-line strategy for searching for the genetic cause of OI.

During a medical and genetic study of the population of the Republic of the North Ossetia Alania, in the village of Kizlyar, Mozdok district, a large Kumyk OI family with 35 members in four generations was revealed. Thirteen OI patients demonstrated highly diverse clinical manifestations.

## 2. Materials and Methods

### 2.1. Patients

The patients were recruited during the expedition of the RCMG specialists to the Republic of the North Ossetia Alania for a genetic and epidemiological study of the local population performed according to the established protocol [16]. A family of the Kumyk ethnic group with 35 members in four generations with a presumptive clinical diagnosis of osteogenesis imperfecta was examined. In total, 33 family members were examined, including 13 patients with various clinical manifestations of OI. The examination was carried out by doctors of various profiles according to the clinical recommendations of the Ministry of Health of the Russian Federation and the Brittle Bones Disease Consortium (BBDC) and Osteogenesis Imperfecta Foundation [8].

### 2.2. Informed Consent

Written informed consent was obtained from each of the 13 participants and/or their legal representative, as appropriate. This study was conducted in accordance with the Declaration of Helsinki and approved by the Institutional Ethics Committee of the Research Centre for Medical Genetics (protocol no. 2017-4/1 dated 4 May 2017).

### 2.3. DNA Extraction

Genomic DNA was extracted from peripheral blood cells using the Promega^®^ DNA Extraction Kit (Promega^®^, Madison, WI, USA) according to the manufacturer’s recommendations.

### 2.4. Sequencing

The patient DNA testing was performed on an NGS sequencer BGISEQ-500 (BGI, Shenzhen, China) by using a paired-end reads method (2 × 100 bp) with mean depth coverage not less than 1971×. The library preparation was accomplished using the selective capture by MGIEasy Exome Capture V4 kit (MGI, Hong Kong, China), encompassing coding regions of the over 20,000 genes (59 Mb).

Data analysis was executed by utilizing an in-house [17] automated algorithm, including sequencing quality control, reads alignment on the human genome reference sequence (GRCh37/hg19), alignment post-processing, and variant calling and filtering by quality, as well as their annotation according to all the established transcripts of RefSeq genes and in silico prediction of the pathogenicity of missense changes by the several algorithms (SIFT, PolyPhen2-HDIV, PolyPhen2-HVAR, MutationTaster, LRT, BMut), and according to the evolutionary conservation score (PhyloP, PhastCons). Allele frequencies in a general population were assessed in gnomAD v.2.1.1 [18]. The obtained technical characteristics of the WES were as follows: raw total sequences: 228076746; total length: 22.8 Gb; total variants called: 177172; total number of variants filtered and assessed according to the pathogenicity criteria as relevant: 1.

Validation of the variant NM_000088.3(*COL1A1*):c.1243C>T p.(Arg415*) in the proband and the variant segregation analysis in the family were carried out using the Sanger sequencing method. The primers used for target sequencing were as follows: 5′- TACTCACGCTGTTACCCTTGG-3′ (19F); 5′- TGCTGTAAGTGTCCCCGACT-3′ (19R).

### 2.5. Methods of Medical Instrumental Examination

The measurements of the height and weight were performed for all patients and healthy family members. The index patient underwent echocardiography (at the age of 5 y.o.), measurement of bone mineral density at the level of the left femur and lumbar spine using an X-ray osteodensitometer (apparatus Stratos DR, DMS Imaging production, A Division of the DMS group, Gallargues-le-Montueux, France) (at the age of 8 y.o.). To determine the status of short stature, a percentile scale was used for compliance with the age and sex references for children and adolescents in the Russian Federation according to the data of the Union of Pediatricians of Russia [7].

## 3. Results

**Figure 1:**
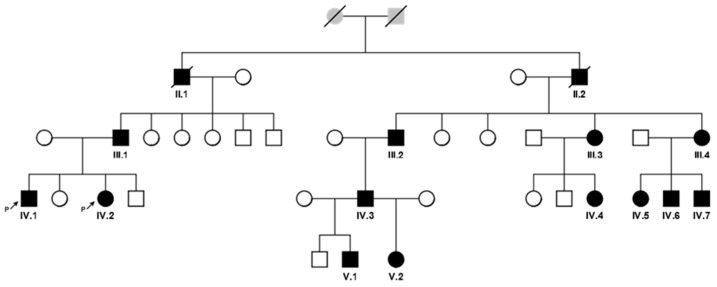
A pedigree of the family with 13 OI patients in 4 generations (affected individuals are colored with black and signed with Roman and Arabic numerals according to the position in the pedigree).

A large Kumyk OI family with 15 patients among 35 members in four generations was revealed. Two patients died by the moment of the study; they were not available for examination and molecular genetic research. A pedigree is shown in Figure 1, the clinical manifestations of the disease in different family members are presented in Table 1.

The clinical diagnosis of OI was considered to be valid in all patients, even if only one of the characteristic features of OI (blue sclera or hearing loss or dentinogenesis imperfecta) was present. The age of the patients examined ranged from 1 year to 54 years, and there were seven men and six women (Table 1). Eleven out of thirteen patients (11/13; 84.62%) had short stature, while healthy family members had normal growth. The examined children at an early age had a delay of motor development for 6–8 months, congenital hypotrophy (the anthropometric measures of the newborns after full-term pregnancies did not exceed 2500-2800/46-49 cm). Also, all patients (13/13; 100%) had blue sclera of various intensity: blue sclera in patients V.I and III.4, dark blue tints in seven patients (III.2, III.3, IV.1, IV.2, IV.3, IV.4, IV.5, IV.6, IV.7. V.2), and blue tints with gray tints in patient III.1. Facial changes, triangular face, and broad forehead were noticeable in the patients in their early childhood.

The number of fractures by the time of examination varied widely. A history of fractures was noted in nine patients (9/13; 69.23%). The first fracture occurred in early childhood, from 12 to 14 months, in all nine patients.

All the pediatric patients (IV.1, IV.2, V.1, V.2, IV.4, IV.5–7) and adults with a large number of fractures (II.1, II.2, III.2, IV.3) underwent X-ray examinations of the spine and tubular bones. None of the examined patients had Wormian ossicles or Codfish vertebrae. However, all patients had signs of osteoporosis/osteopenia and traces of compression fractures of the spine. Also, during multiple laboratory studies, no changes in the alkaline phosphatase level were found in index patient IV.1 (214 U/L), in his sister IV.2 (170 U/L), or in their cousin IV.4 (261 U/L) and her mother III3 (145 U/L), while a decreased level of this indicator was noted in siblings V.1, V.2 (170 and 182 U/L), and their father IV.3 (56 U/L). Patient IV.4 had decreased bone mineral density (−3.1 SD according to the Z-score) and a large number of fractures. Patients V.1 and V.2 also showed a significant decrease in bone mineral density (−1.8 and −2.0 SD according to the Z-score), but did not have fractures over the previous 2 years.

To identify the genetic cause of the disease in a described family, whole-exome sequencing using NGS technology was performed in proband IV.1. The proband, a 9-year-old boy, demonstrated short stature, <3 percentile, and 10 tubular bone fractures; the first fracture occurred at the age of 14 months, without bone deformities, scoliosis of grade 2, flat feet, dentinogenesis imperfecta manifested as extensive caries, and blue sclera (Table 1). At an early age, the proband had a 6-month motor development delay and congenital hypotrophy (with a full-term pregnancy, the anthropometric parameters of the newborn did not exceed 2500 g and 49 cm). Electrocardiography revealed the manifestation of cardiac signs of connective tissue dysplasia; mitral regurgitation was detected at the age of 5 years. Densitometry of the spine at the level of L1-L4 and the left hip (average for the neck, greater trochanter, and intertrochanteric space) revealed reduced bone mineralization (Z score −2.5).

**Figure 2:**
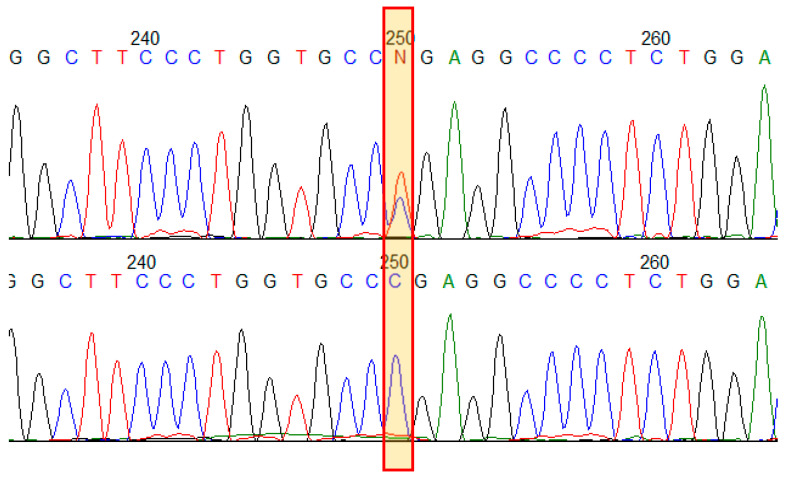
Results of Sanger sequencing of the *COL1A1* gene exon 19 in the family. Electropherograms demonstrate the variant NM_000088.3(*COL1A1*):c.1243C>T p.(Arg415Ter) in a heterozygous state in an affected family member (**upper**) and in a healthy one (**bottom**).

The proband was found to have a known pathogenic variant (HGMD:CM960321) in exon 19 (out of 51) of the *COL1A1* gene: NM_000088.3(*COL1A1*):c.1243C>T, in a heterozygous state, leading to the formation of a premature termination codon (PTC) p.(Arg415Ter). The variant was not found among healthy people (gnomAD v2.1.1) [18]. In silico analysis shows that aberrant mRNA does not escape nonsense-mediated decay [19]. That is a loss of the function variant (LoF) in the *COL1A1* gene, which is intolerant to LoF mutational changes; the variant has a deleterious functional impact. COL1A1 function haploinsufficiency due to LoF variants presents a uniform mechanism of OI type I [20]. The variant appears to be recurrent and the most frequent among 1456 known *COL1A1* pathogenic variants; it has been reported at least 20 times in patients with OI of different types [10]. No other variants in relevant genes were found. To verify NGS finding and to analyze segregation of the variant in the family with 13 presumably affected relatives with various phenotypic features of OI and several available healthy family members, Sanger sequencing of exon 19 of the *COL1A1* gene was performed (Figure 2). All 13 patients were found to have the variant NM_000088.3(*COL1A1*):c.1243C>T in a heterozygous state. The variant was not found in healthy family members.

## 4. Discussion

### 4.1. Clinical Polymorphism

A family of Kumyk origin with osteogenesis imperfecta was consulted during a genetic and epidemiological study of the population of the Republic of the North Ossetia Alania [16]. Thirteen patients were identified among 35 family members in four generations. Two more deceased people were also known to be affected. In the index patient, a known pathogenic variant in the *COL1A1* gene NM_000088.3(*COL1A1*):c.1243C>T, leading to the formation of a premature translation termination codon p.(Arg415*) in a heterozygous state, was identified by NGS technology. The variant in a heterozygous state was found in all 13 affected family members and was not found in healthy ones; no other variants in relevant genes were found. The variant c.1243C>T in a heterozygous state was previously described in patients with osteogenesis imperfecta types I [21,22], III [12], and Ehlers–Danlos syndrome [23]. In the examined family, the variant led to OI type IV.

**Figure 3:**
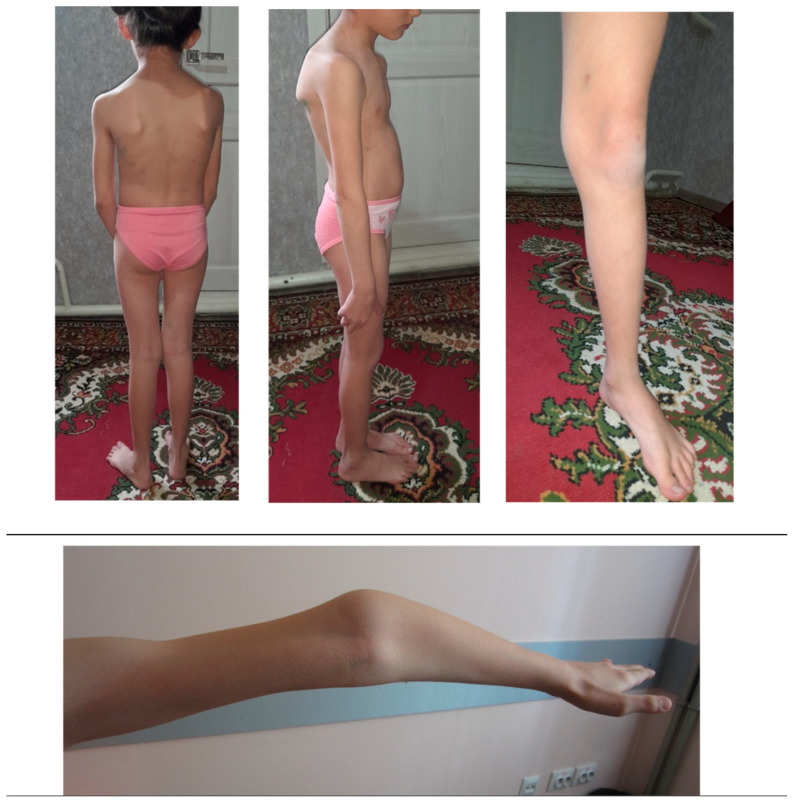
Patient IV.4, a girl of 8 y.o., with severe scoliosis, flat feet, and joint hypermobility.

In some cases, the clinical manifestation of OI may differ from the expected one, which can be explained by mosaicism due to a de novo mutation. In other cases, it may be a coincidence of two or more pathogenic variants in the same patient. [24]. Nevertheless, even without mosaicism or other pathogenic genetic variants, the variety of OI symptoms within one family can be considerable [11]. The clinical variability of OI has not yet been fully explained. Though descriptive analysis was not possible due to a small number of patients of the same age in each nuclear family, distinct intrafamilial polymorphism of OI symptoms and their severity was observed. For example, child IV.5 (at the time of examination at 1 year) had only one symptom (dark blue sclerae). Other patients showed a more extended picture of the disease. Patient IV.4, a girl of 8 y.o., demonstrated severe scoliosis, flat feet, and joint hypermobility (Figure 3).

The most pronounced clinical picture of the disease with the most numerous symptoms was found in patient III.1, a man of 44 y.o. He had a short stature, 15 long bone fractures, long bone deformity, dentinogenesis imperfecta, blue sclerae, and conductive hearing loss. In Table 1, the patients’ data are presented in clusters according to the eight nuclear families they belong to. While grouping the patients according to age, one could notice that the OI phenotype’s severity may correspond with the age. The number and proportions of the patients with different clinical signs from the three age groups are presented in Table 2.

Distinguishing at least three age groups, one with infants from 1 to 3 years old (N = 4, IV.5, IV.2. V.2, IV.6), the second with children from 6 to 9 years old (N = 4, IV.7, IV.4, IV.1, V.I), and the third with adults from 24 years old and older (N = 5, III.4, III.3, III.1, III.2, IV.3), could demonstrate the following patterns. No one in the first group had fractures or bone deformities, although all four had blue sclera, two of them had delayed tooth eruption, and three had FTT (failure to thrive); in the second group, each of the four child patients had several fractures, bone deformities, and signs of connective tissue dysplasia, along with blue sclera; one child had signs of DI, and three had FTT; in the third age group each adult had multiple fractures and bone deformities, as well as blue sclera of different shades and signs of DI. Thus, at least a part of the clinical diversity within a large family could be explained by the age, as symptoms of OI develop in relation to the age. On the one hand, the number of fractures could be accrued with age; on the other hand, the clinical course of OI could improve after ~55 years old. [25].

Theoretically, belonging to different nuclear families could also play a role in the set of manifestations and severity of the disease and explain some part of observed clinical variability. Some similarities within the family clusters could be inferred; e.g., the alkaline phosphatase level was normal in girl IV.4 and her mother III.3, and decreased in siblings V.1, V.2, and their father IV.3. Decreased bone mineral density was either associated with a large number of fractures (patient IV.4) or it was not. Siblings V.1 and V.2 had decreased bone mineral density but neither had had fractures over the previous 2 years. The clinical picture similarity inside the nuclear families and the differences between them seem to be not clear.

Additional genetic, epigenetic, and environmental factors are proposed to alter the clinical manifestations of OI in different families and in individuals. Modifying genetic factors could concern the COL1A1 molecular landscape and include other genes from the pathways of procollagen I or collagen I processing and transporting [26], as well as osteoblast differentiation maintenance and function [27]. Micro-RNAs and other epigenetic regulations of the same pathways could theoretically affect the severity of manifestations of OI [28]. Environmental influences, including micronutrient consumption, could also make their contribution to the OI phenotype [29]. A possible micronutrient disbalance impact has been shown recently for different types of OI. Trace elements and minerals are important not only for normal bone structure and mineralization but also for function as they present key pathway regulators in the osteoblasts and osteoclasts [29,30]. Also, intrafamilial diversity potential was found to depend on the genotype characteristics of the OI-causing pathogenic variants. Statistical analysis of the phenotypic diversity associated with various *COL1A1* variants has revealed that the variant c.1243C>T p.(Arg415*) is linked to increased intrafamilial variation [31]. The modifying factors can significantly impact the clinical manifestation of OI. They are being actively studied worldwide in order to find effective treatments for OI. However, at the moment, we can only speculate about their influence.

### 4.2. Most Common Signs of OI in the Family

The diagnosis is valid, as in all the age groups from different nuclear families, almost all the patients (11/13) had FTT and short stature, as well as blue sclera. Approximately half of the patients from all the age groups from different nuclear families had DI. Fractures and deformities were present in all the adult patients from different nuclear families. The manifestation of OI clinical symptoms widely varied within one family, and yet, the characteristic moderate short stature, varying degrees of scoliosis, and blue, grayish, or white sclerae, as well as signs of DI, allow us to suggest the diagnosis of OI type IV in the family.

### 4.3. The Variant NM_000088.3(COL1A1):c.1243C>T p.(Arg415*)

According to the literature, clinical manifestations of the variant c.1243C>T p.(Arg415*) are different in different families. Table 3 presents clinical data of the patients who were heterozygous for the variant NM_000088.3(*COL1A1*):c.1243C>T p.(Arg415*) available from the literature and of the index patient IV.1 from this study.

The patients with the variant showed a wide range of OI symptom severity and a stature from normal to short; two of them had blue sclerae, and all of them had different numbers of recurrent fractures on minor trauma, from 1 to over 10. The spectrum of associated manifestations varied significantly; diagnoses ranged from Ehlers–Danlos syndrome and the mildest form of OI, type I, to a rather severe form of OI, type III.

The association of Ehlers–Danlos syndrome (EDS) with variants in the *COL1A1* and *COL1A2* genes was established earlier [20,23,32]. There are three different EDS types associated with pathogenic variants in the *COL1A1* or *COL1A2* genes: classical EDS with arterial fragility, arthrochalasis EDS, and cardiac valvular EDS [33]. In the study, some patients presented with severe clinical manifestations, including a high number of fractures and short stature, and were diagnosed with OI type IV; the others had less severe OI and/or EDS features.

The variant c.1243C>T p.(Arg415*) results in the formation of a premature termination codon and loss of half of the normal COL1A1 open reading frame encoding the α1 chain of procollagen [21]. Functional analysis shows that this and other monoallelic loss-of-function (LoF) variants of the *COL1A1* gene result only in a quantitative decrease in normal mRNA, since the aberrant RNA is degraded by a nonsense-mediated mechanism. LoF *COL1A1* variants realize a haploinsufficiency mechanism, leading to less severe impairment of type 1 collagen function and the development of OI type I [21], though phenotypes of the patients with the variant could differ from OI type I [12,20]. *COL1A1* missense variants that result in a glycine substitution in the α chain region involved in triple-helix formation or that alter proteolytic sites in the N or C termini of procollagen flanking the triple-helix domain result in collagen structural changes, worse functional consequences, and a more severe phenotype in patients. They are thought to have a dominant-negative effect [34] and to lead to OI types II, III, or IV.

### 4.4. Treatment

The molecular mechanism of OI has not been sufficiently studied. Collagen 1 is the main organic component of the bone matrix. Collagen 1 is synthesized by osteoblasts and fibroblasts and undergoes important post-translational modifications in the endoplasmic reticulum. It assembles into a stable triple-helix structure, and these procollagen chains are excreted into the intercellular matrix. Regulation of osteoblast and osteoclast function underlies the methods of treatment of OI that are being developed and are already used [35]. Bisphosphonates have been successfully used in the treatment of children with autosomal dominant OI since the 1980s and remain the standard of care for OI. They are administered with a preliminary course of calcium and vitamin D. Bisphosphonates destroy the key pathway of osteoclast metabolism regulation, leading to suppression of their function and apoptosis [35]. This study revealed a dominant form of OI caused by the LoF variant in a heterozygous state in the *COL1A* gene. This form responds well to bisphosphonate treatment in children, and such treatment can significantly improve the quality of life of patients and the entire affected family [35].

The “golden standard” therapy with zoledronic acid was performed for one patient from the study. Therapy indications took into account the severity of the disease, the frequency of fractures, the degree of bone mineral density impairment (osteopenia/osteoporosis), the presence of skeletal deformities, and delayed physical development. Bisphosphonate therapy is not indicated for patients with type I OI, characterized by a mild course (less than three fractures in the last year, no deformities of long tubular bones, and no delayed physical development). A year ago, children IV.4, V.1, and V.2 underwent a complete paraclinical examination with subsequent determination of treatment tactics. According to the results of densitometry (total body), patient IV.4 had a significant decrease in bone mineral density (−3.1 SD according to the Z-score). Considering that and episodes of multiple fractures, it was recommended to initiate therapy with zoledronic acid at an initial dose of 0.0125 mg/kg, once, intravenously by drip (dose 0.16 mg) and a subsequent intake of calcium preparations of 1000 mg/day with a subsequent gradual reduction in the dose within a month and vitamin D3 1000 IU per day for a year. According to the results of densitometry (total body), patients V.1 and V.2 also showed a significant decrease in bone mineral density (−1.8 and −2.0 SD according to the Z-score, respectively), but given the absence of fractures over the previous 2 years and normalization of parathyroid hormone levels while taking cholecalciferol at a dosage of 2000 IU/day, it was decided that the children did not need therapy with zoledronic acid drugs. Subsequently, all the patients received cholecalciferol drugs at a dosage of 2000 IU/day and calcium drugs at 500 mg per day continuously. Over the past year, patient IV.4 has shown increased physical activity and endurance, a height gain of +4 cm, and no fractures even against the background of injuries and falls. Patient V.1 is active, goes in for swimming, and has gained 3 cm in height; a fracture of the metacarpal bones of the left hand occurred against the background of an injury. Patient V.2 also shows positive dynamics; the girl grew by 3.5 cm, is active, practices therapeutic exercise, and has no fractures.

## 5. Conclusions

Clinical manifestations of OI can vary widely within and between families. Patients with mild manifestations might not be detected by clinicians unless the entire OI family is examined. In isolated cases with an incomplete clinical picture in the absence of molecular genetic testing, the complexity of clinical diagnosis is obvious. Confirmation of the diagnosis of osteogenesis imperfecta requires a combination of clinical and molecular genetic methods and the examination of all available family members. In addition, genetic information is important for correct medical genetic counseling to determine the risk of asymptomatic or mildly affected patients having a severely affected offspring. Even if the spectrum of symptoms and the severity of the phenotype in OI are often similar in different patients, the distribution of patients into different types of the disease can be useful in assessing the prognosis of the disease, choosing a treatment method, and predicting the effect of treatment.

## Figures and Tables

**Table 1:** Clinical diversity of OI symptoms among the examined patients.

Patient ID	Age (Years Old)	Sex, Age and Age, Gender Standards for Height and Weight (cm, kg)	Height/Weight, Failure to Thrive (FTT)/Hypotrophy	Fractures (N)	Deformity, Grade and Site	Connective Tissue Dysplasia	Dentinogenesis Imperfecta (DI)	Sclerae Tints	Bone Mineral Density
V.I	9	Male (131–142, 25.2–32.6)	146/26, Tall stature/Norm	N = 21	Moderate, forearm	Dolichostenomelia	Norm	Blue sclerae	(−1.8 SD according to the Z-score)
V.2	3	Female (92–99, 12.7–16.2)	89/12 FTT/Hypotrophy	Without fractures	Without deformities	Joint hypermobility	Norm	Dark blue sclerae	(−2.0 SD according to the Z-score)
IV.1	9	Male (131–142, 25.2–32.6)	120/26 FTT/Norm	N = 10	Without deformities	Scoliosis (2 grade, right side), flatfeet	DI (extensive dental caries)	Blue sclerae	(−2.5 SD according to the Z-score)
IV.2	1.5	Female (84–90, 11–14.2)	69/9 FTT/Hypotrophy	Without fractures	Without deformities	Recurred dislocation of shoulder and elbow joints	Delayed eruption	Dark blue sclerae	n/t
IV.3	29	Male (164–185)	145 FTT	N = 20	Moderate, forearm	Norm	Norm	Dark blue sclerae	n/t
IV.4	8	Female (121–132, 22.1–29.5)	110/19 FTT/Hypotrophy	N = 6	Moderate, cnemis	Scoliosis (2 grade, right side), flatfeet	Norm	Dark blue sclerae	(−3.1 SD according to the Z-score)
IV.5	1	Female (70–78, 8–11.5)	Norm/Norm	Without fractures	Without deformities	Norm	Delayed eruption	Dark blue sclerae	n/t
IV.6	3	Female (97–194, 13.8–17.2)	86/14 FTT/Norm	Without fractures	Without deformities	Joint hypermobility	Norm	Dark blue sclerae	n/t
IV.7	6	Female (116–125, 19–24.5)	101/14 FTT/Hypotrophy	N = 4	Without deformities	Recurred dislocation of shoulder joint	Norm	Dark blue sclerae	n/t
III.1	44	Male (164–185)	158 FTT	N = 15	Slight, forearm, cnemis, knee joints	Norm	Prosthodontics from 30 y.o, DI	Dark blue with grayish tint sclerae	n/t
III.2	54	Male (164–185)	156 FTT	N = 30	Moderate, forearm	Norm	Prosthodontics from 18 y.o, DI	Dark blue sclerae	n/t
III.3	28	Female (158–180)	144 FTT	N = 7	Moderate, forearm	Norm	Norm	Dark blue sclerae	n/t
III.4	24	Female (158–180)	151 FTT	N = 5	Without deformities	Norm	Norm	Blue sclerae	n/t
II.1 (*)	Deceased	Male	Short stature	N = 20, n/t	Moderate, cnemis	n/t	Early loss of a tooth	n/t	n/t
II.2 (*)	Deceased	Male	Short stature	N = 10, n/t	n/t	n/t	Early loss of a tooth	n/t	n/t

**Note**: For the people older than 18 y.o., only height standard accordance/discordance was under consideration; n/t—no information, not tested; *—dead people, unavailable for examination and molecular genetic testing, fulfilled according to the health care’s words.

**Table 2:** Clinical signs of patients in 3 different age groups (within the same large family).

Sign of Osteogenesis Imperfecta	Number and Share of the Patients with Clinical Feature (Group I)	Number and Share of the Patients with Clinical Feature (Group II)	Number and Share of the Patients with Clinical Feature (Group III)	Total Number and Share of the Patients with Clinical Feature in the Family
Blue sclerae	4/4	4/4	5/5	13/13 (100%)
Failure to thrive (FTT)/short stature	3/4	3/4	5/5	11/13 (85%)
Dentinogenesis imperfecta	2/4	1/4	2/5	5/13 (38%)
Fractures	0/4	4/4	5/5	9/13 (69%)
Deformations	0/4	2/4	5/5	7/13 (54%)
Recurred dislocation of joints	1/4	1/4	0/5	2/13 (15%)
Scoliosis	0/4	2/4	0/5	2/13 (15%)
Joint hypermobility	2/4	0/4	0/5	2/13 (15%)
Dolichostenomelia	0/4	1/4	0/5	1/13 (8%)
Conductive deafness	0/4	0/4	1/5	1/13 (8%)

**Table 3:** Clinical manifestations of OI in the patients heterozygous for the variant c.1243C>T p.(Arg415*) according to literature data and in the index patient in this study.

Patients	Sex	Years	Height	Skeletal Anomalies	Non-Skeletal Anomalies	Other Features (Connective Tissue Dysplasia)
Patient with Osteogenesis imperfecta I [21]	unknown	unknown	Short stature (Each affected individual met the clinical criteria for type I)	Recurrent fractures on minor trauma	Blue sclerae	n/t
Patient with Osteogenesis imperfecta IA [22]	M	22	Normal stature	Lower incidence of fractures	n/t	n/t
Patient with Osteogenesis imperfecta III [12]	M	3	Short stature	Recurrent fractures on minor trauma	n/t	n/t
Patient with Ehlers–Danlos syndrome [20]	F	23	Normal	1 long bone fracture, long bone deformity, joint pain, flatfeet	Blue sclerae	Neonatal hypotonia, soft, doughy skin, piezogenic papules
Index patient from this study	M	9	Short stature	10 long bone fractures, low bone density	Blue sclerae DI (extensive dental caries)	Mitral regurgitation

Note. DI—Dentinogenesis Imperfecta, n/t—not tested, no information available.

## Data Availability

The original contributions presented in this study are included in the article. Further inquiries can be directed to the corresponding author.
